# 4-[(4-Fluoro­phen­yl)amino]-5-(2-meth­oxy­phen­yl)-8,8-dimethyl-6,7,8,9-tetra­hydro-5*H*-chromeno[2,3-*d*]pyrimidin-6-one

**DOI:** 10.1107/S2414314626010461

**Published:** 2026-10-30

**Authors:** Adesola A. Adeleke, Sizwe J. Zamisa, Orpah Zinyemba, Bernard Omondi, Olatunde S. Olatunji

**Affiliations:** a School of Agriculture and Science, Discipline of Chemistry, University of KwaZulu-Natal, Private Bag X54001, Durban, 4000, South Africa; b Department of Chemical Sciences, Olabisi Onabanjo University, Ago-Iwoye, Nigeria; c Department of Chemical Sciences, University of Johannesburg, PO Box 524 Auckland, Park, Johannesburg, 2006, South Africa; Katholieke Universiteit Leuven, Belgium

**Keywords:** crystal structure, chromeno­pyrimidine

## Abstract

The asymmetric unit of the title compound, C_26_H_24_FN_3_O_3_, contains one mol­ecule comprising a tricyclic chromeno[2,3-*d*]pyrimidine core formed by fused cyclo­hexa­none, pyran and pyrimidine rings. The *sp*^3^-hybridized methine carbon atom bears a 2-meth­oxy­phenyl substituent, which is oriented almost perpendicular to the pyran ring, with a dihedral angle of 84.11 (5)°. The 4-fluoro­phenyl­amino substituent is twisted relative to the pyrimidine ring, as indicated by the C—C—N—C torsion angle of 29.79 (18)° and the dihedral angle of 27.59 (4)° In the crystal, C—H⋯O and C—H⋯F hydrogen bonds link the mol­ecules into supra­molecular chains extending diagonally within the *ac* plane. An intra­molecular N—H⋯O hydrogen bond is also present between the amino and meth­oxy groups.

## Abstract 2

The title chromeno[2,3-*d*]pyrimidine derivative forms a supra­molecular chain in the crystal.

## Graphical abstract

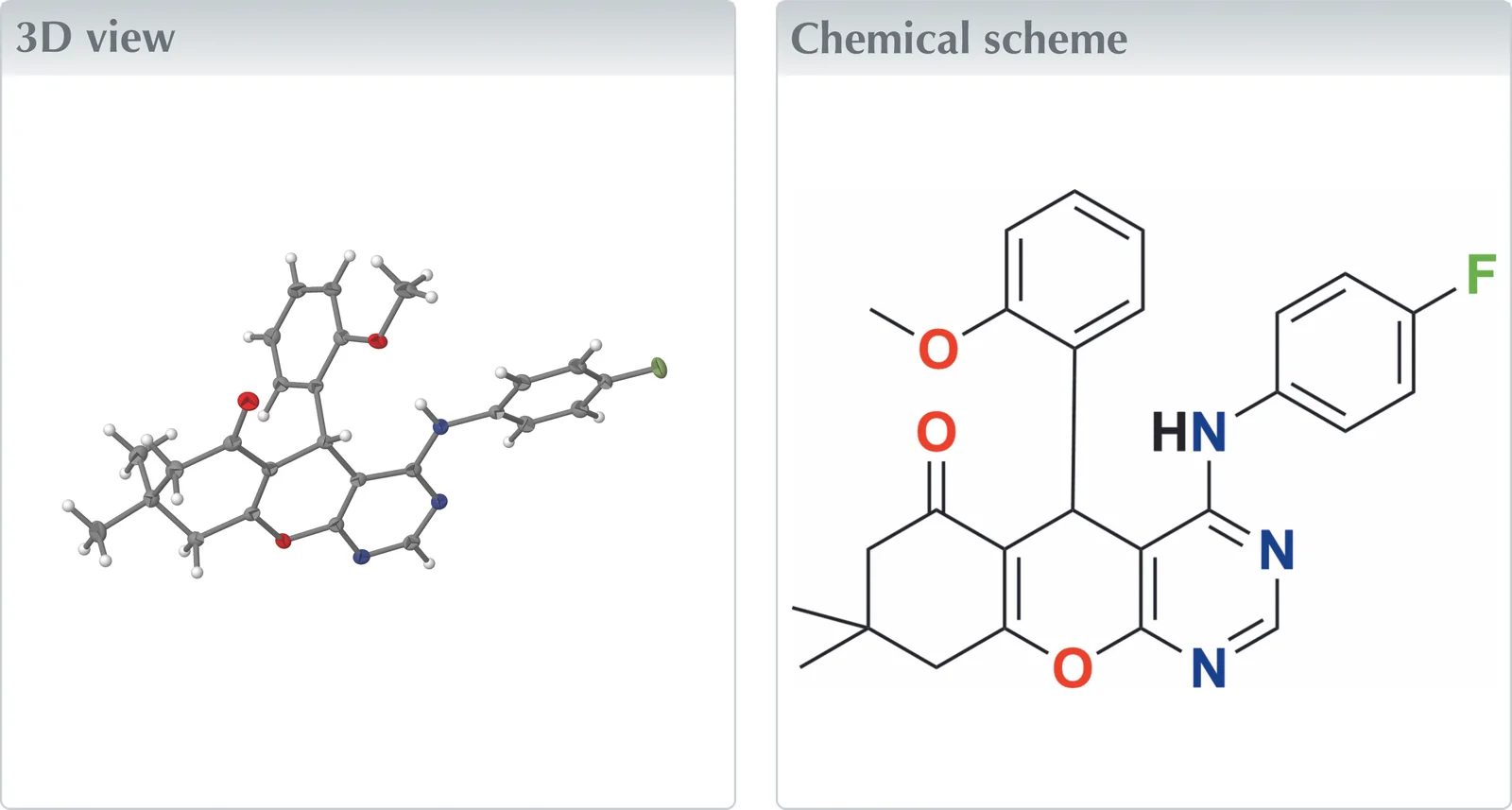

## Structure description

Fused chromeno[2,3-*d*]pyrimidine frameworks constitute an important class of heterocyclic systems in medicinal chemistry because they combine the pharmacologically privileged chromene and pyrimidine motifs within a single structure. Members of this family have been reported to display a wide range of biological effects, including anti­proliferative, anti­microbial, anti­viral, anti-inflammatory and enzyme-inhibitory activities (Patil, 2023 ▸; Zamisa *et al.*, 2023 ▸; Noser *et al.*, 2026 ▸), which has stimulated extensive synthetic and structural work on their derivatives. Tetra­hydro­chromeno[2,3-*d*]pyrimidin-6-one scaffolds bearing *N*-aryl amino substituents on the pyrimidine ring and aryl groups at the methine centre of the pyran moiety have been associated with enhanced cytotoxicity and improved DNA/BSA binding properties, as demonstrated in recent structure-activity and biophysical studies on related systems (Zamisa *et al.*, 2023 ▸; Noser *et al.*, 2026 ▸). As part of an ongoing study into the structural chemistry of this scaffold, we report here the crystal structure of the title compound, C_26_H_24_FN_3_O_3_.

The asymmetric unit contains one mol­ecule (Fig. 1 ▸) and the mol­ecule features a tricyclic chromeno[2,3-*d*]pyrimidine core comprising three fused rings: the cyclo­hexa­none, the pyran­oid, and the pyrimidine (Zamisa *et al.*, 2023 ▸). The presumed *sp*^3^-hybridized carbonatom C11 bears a pendant 2-meth­oxy­phenyl substituent and adopts an almost perpendicular orientation with respect to the pyran­oid ring [dihedral angle = 84.11 (5)°]. In contrast, the 4-fluoro­phenyl­amino moiety appears to be twisted with respect to the pyrimidine moiety [torsion angle C21—C20—N3—C9 = 29.79 (18)°]. Other geometrical data appear to be comparable with those of related compounds (Zamisa *et al.*, 2023 ▸). In the crystal, C—H⋯O and C—H⋯F hydrogen bonds form supra­molecular chains extending diagonally along the crystallographic *ac* plane (Fig. 2 ▸). Intra­molecular N—H⋯O hydrogen bonding is observed between the H3 and O3 atoms of the amine and meth­oxy groups, respectively (Table 1 ▸).

## Synthesis and crystallization

A mixture of ethyl (*E*)-*N*-(3-cyano-4-(2-meth­oxy­phen­yl)-7,7-dimethyl-5-oxo-5,6,7,8-tetra­hydro-4*H*-chromen-2-yl)formimidate (1.0 mmol) was combined with 4-fluoro­aniline (1.2 mmol) in glacial acetic acid (10 ml), sealed in a 30 ml microwave vial and irradiated at 200 W for 20 min. Reaction completion was monitored by TLC. Distilled water was added to the hot mixture without stirring, allowed to stand overnight, and the resulting precipitate was collected by vacuum filtration, washed with water and recrystallized from a mixed solvent of ethanol and water. Colourless block-shaped single crystals suitable for X-ray diffraction were obtained by slow evaporation of a methano­lic solution at room temperature.

## Refinement

Crystal data, data collection and structure refinement details are summarized in Table 2 ▸.

## Supplementary Material

Crystal structure: contains datablock(s) I. DOI: 10.1107/S2414314626010461/vm4087sup1.cif

Structure factors: contains datablock(s) I. DOI: 10.1107/S2414314626010461/vm4087Isup2.hkl

Supporting information file. DOI: 10.1107/S2414314626010461/vm4087Isup3.cml

CCDC reference: 2594290

Additional supporting information:  crystallographic information; 3D view; checkCIF report

## Figures and Tables

**Figure 1:**
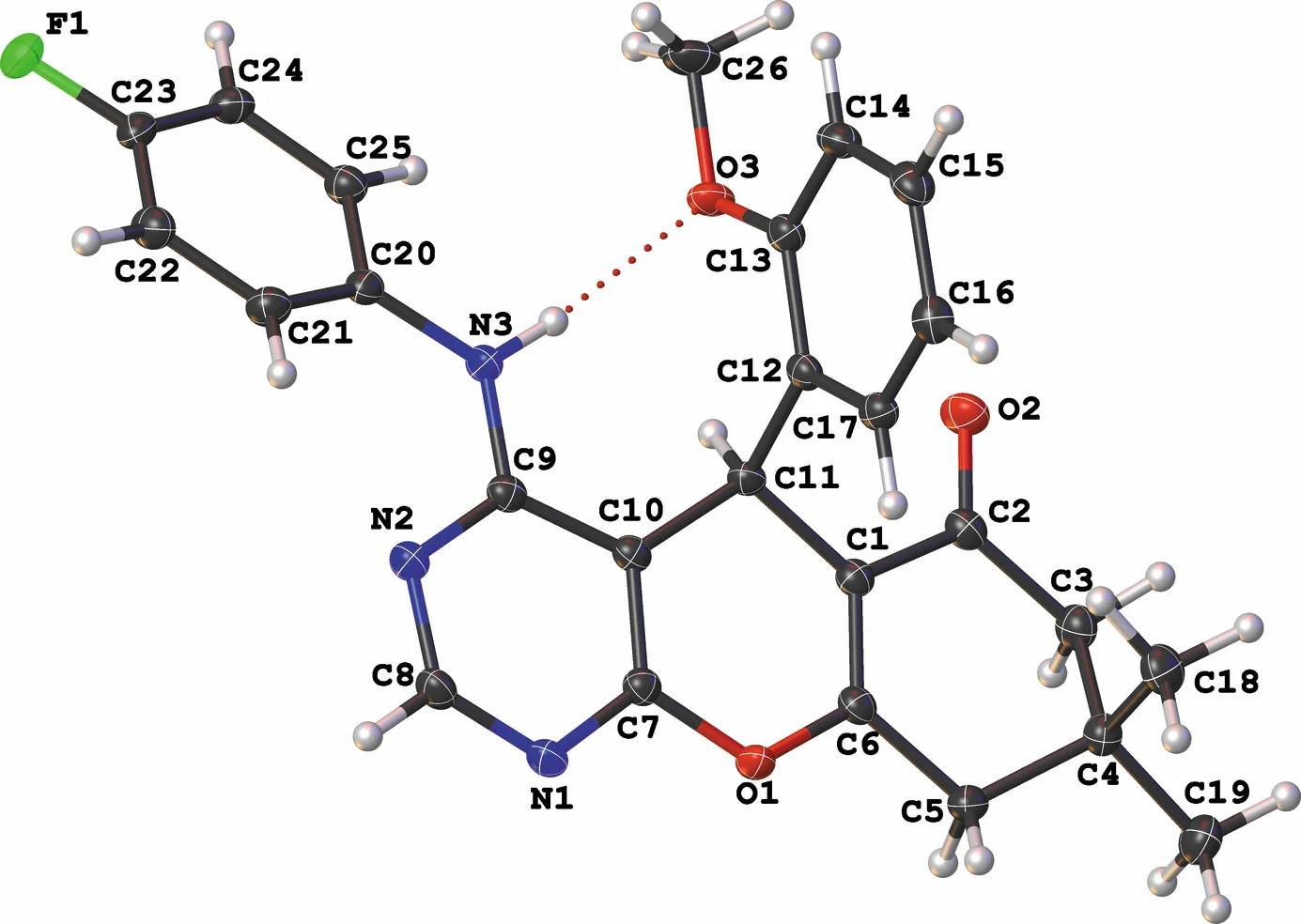
Mol­ecular structure of the title compound with ellipsoids drawn at the 50% probability level.

**Figure 2:**
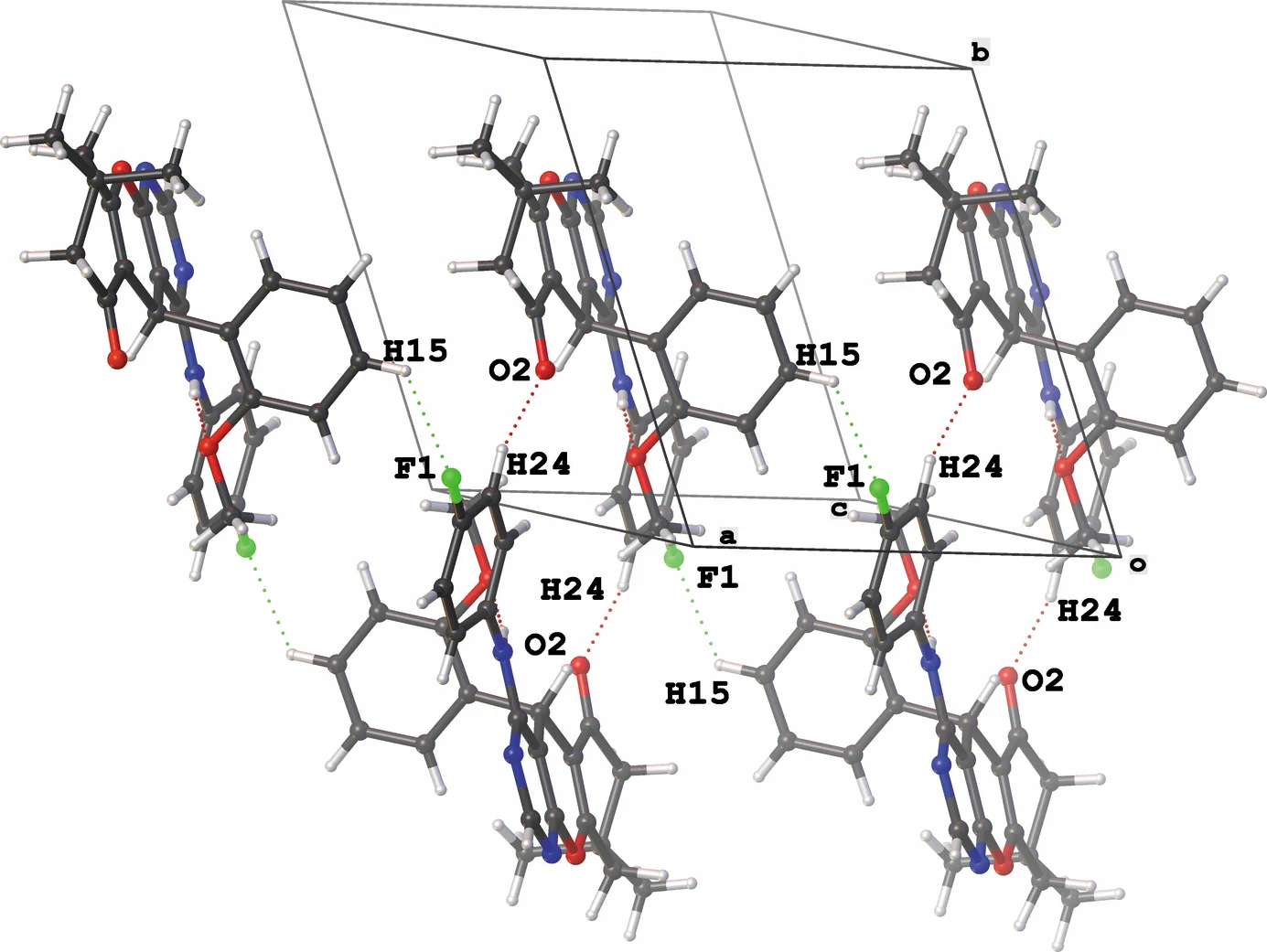
Representation of the inter­molecular C—H⋯O (red dotted lines) and C—H⋯F (green dotted lines) hydrogen bonds in the crystal packing of the title compound.

**Table 1:** Hydrogen-bond geometry (Å, °)

*D*—H⋯*A*	*D*—H	H⋯*A*	*D*⋯*A*	*D*—H⋯*A*
N3—H3⋯O3	0.88	2.08	2.9579 (13)	172
C15—H15⋯F1^i^	0.95	2.62	3.2998 (16)	129
C24—H24⋯O2^ii^	0.95	2.39	3.1930 (16)	141

Symmetry codes: (i) 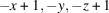; (ii) 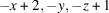.

**Table 2:** Experimental details

Crystal data	
Chemical formula	C_26_H_24_FN_3_O_3_
*M*_r_	445.48
Crystal system, space group	Triclinic, *P* 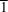
Temperature (K)	100
*a*, *b*, *c* (Å)	9.1806 (3), 10.3535 (3), 12.8852 (4)
α, β, γ (°)	66.585 (1), 88.943 (1), 78.562 (1)
*V* (Å^3^)	1099.09 (6)
*Z*	2
Radiation type	Mo *K*α
μ (mm^−1^)	0.10
Crystal size (mm)	0.28 × 0.16 × 0.12

Data collection	
Diffractometer	Bruker SMART APEXII area detector
Absorption correction	Multi-scan (*SADABS*; Krause *et al.*, 2015 ▸))
*T*_min_, *T*_max_	0.964, 0.996
No. of measured, independent and observed [*I* > 2σ(*I*)] reflections	25611, 4509, 4110
*R*_int_	0.018
(sin θ/λ)_max_ (Å^−1^)	0.626

Refinement	
*R*[*F*^2^ > 2σ(*F*^2^)], *wR*(*F*^2^), *S*	0.034, 0.091, 1.04
No. of reflections	4509
No. of parameters	301
H-atom treatment	H-atom parameters constrained
Δρ_max_, Δρ_min_ (e Å^−3^)	0.31, −0.23

Computer programs: *APEX2*,*COSMO* and *SAINT* (Bruker, 2009 ▸), *SHELXT* (Sheldrick, 2015*a* ▸), *SHELXL2018/3* (Sheldrick, 2015*b* ▸) and *OLEX2* (Dolomanov *et al.*, 2009 ▸).

## full crystallographic data

### 4-[(4-Fluorophenyl)amino]-5-(2-methoxyphenyl)-8,8-dimethyl-6,7,8,9-tetrahydro-5H-chromeno[2,3-d]pyrimidin-6-one Crystal data

**Table:**

C_26_H_24_FN_3_O_3_	*Z* = 2
*M**_r_* = 445.48	*F*(000) = 468
Triclinic, *P*1	*D*_x_ = 1.346 Mg m^−^^3^
*a* = 9.1806 (3) Å	Mo *K*α radiation, λ = 0.71073 Å
*b* = 10.3535 (3) Å	Cell parameters from 9909 reflections
*c* = 12.8852 (4) Å	θ = 2.8–26.4°
α = 66.585 (1)°	µ = 0.10 mm^−^^1^
β = 88.943 (1)°	*T* = 100 K
γ = 78.562 (1)°	Block, colourless
*V* = 1099.09 (6) Å^3^	0.28 × 0.16 × 0.12 mm

### 4-[(4-Fluorophenyl)amino]-5-(2-methoxyphenyl)-8,8-dimethyl-6,7,8,9-tetrahydro-5H-chromeno[2,3-d]pyrimidin-6-one Data collection

**Table:**

Bruker SMART APEXII area detector diffractometer	4509 independent reflections
Radiation source: microfocus sealed X-ray tube, Incoatec Iµs	4110 reflections with *I* > 2σ(*I*)
Mirror optics monochromator	*R*_int_ = 0.018
Detector resolution: 7.9 pixels mm^-1^	θ_max_ = 26.4°, θ_min_ = 1.7°
ω and φ scans	*h* = −11→11
Absorption correction: multi-scan (SADABS; Krause *et al.*, 2015))	*k* = −12→12
*T*_min_ = 0.964, *T*_max_ = 0.996	*l* = −16→16
25611 measured reflections	

### 4-[(4-Fluorophenyl)amino]-5-(2-methoxyphenyl)-8,8-dimethyl-6,7,8,9-tetrahydro-5H-chromeno[2,3-d]pyrimidin-6-one Refinement

**Table:**

Refinement on *F*^2^	Primary atom site location: dual
Least-squares matrix: full	Hydrogen site location: inferred from neighbouring sites
*R*[*F*^2^ > 2σ(*F*^2^)] = 0.034	H-atom parameters constrained
*wR*(*F*^2^) = 0.091	*w* = 1/[σ^2^(*F*_o_^2^) + (0.0443*P*)^2^ + 0.4296*P*] where *P* = (*F*_o_^2^ + 2*F*_c_^2^)/3
*S* = 1.04	(Δ/σ)_max_ < 0.001
4509 reflections	Δρ_max_ = 0.31 e Å^−^^3^
301 parameters	Δρ_min_ = −0.23 e Å^−^^3^
0 restraints	

### 4-[(4-Fluorophenyl)amino]-5-(2-methoxyphenyl)-8,8-dimethyl-6,7,8,9-tetrahydro-5H-chromeno[2,3-d]pyrimidin-6-one Special details

**Table:**

Geometry. All esds (except the esd in the dihedral angle between two l.s. planes) are estimated using the full covariance matrix. The cell esds are taken into account individually in the estimation of esds in distances, angles and torsion angles; correlations between esds in cell parameters are only used when they are defined by crystal symmetry. An approximate (isotropic) treatment of cell esds is used for estimating esds involving l.s. planes.

### 4-[(4-Fluorophenyl)amino]-5-(2-methoxyphenyl)-8,8-dimethyl-6,7,8,9-tetrahydro-5H-chromeno[2,3-d]pyrimidin-6-one Fractional atomic coordinates and isotropic or equivalent isotropic displacement parameters (Å^2^)

**Table:**

	*x*	*y*	*z*	*U*_iso_*/*U*_eq_	
F1	0.51611 (8)	−0.12289 (7)	0.93693 (6)	0.02766 (17)	
O1	0.88026 (9)	0.71158 (8)	0.33977 (6)	0.01804 (17)	
O2	1.08883 (9)	0.33314 (9)	0.23063 (7)	0.02328 (19)	
O3	0.83643 (9)	0.13873 (8)	0.40667 (7)	0.01960 (18)	
N1	0.74030 (11)	0.69456 (10)	0.48757 (8)	0.0184 (2)	
N2	0.65665 (11)	0.47975 (10)	0.60063 (8)	0.0184 (2)	
N3	0.72526 (10)	0.27245 (10)	0.56532 (8)	0.0174 (2)	
H3	0.766657	0.235970	0.518106	0.021*	
C1	0.97487 (11)	0.51445 (11)	0.28823 (9)	0.0151 (2)	
C2	1.07612 (12)	0.45792 (12)	0.21895 (9)	0.0174 (2)	
C3	1.16585 (12)	0.55736 (12)	0.13772 (10)	0.0202 (2)	
H3A	1.187618	0.528121	0.073716	0.024*	
H3B	1.262168	0.545493	0.177245	0.024*	
C4	1.08871 (12)	0.71698 (12)	0.09010 (9)	0.0190 (2)	
C5	1.04334 (12)	0.75641 (12)	0.19102 (9)	0.0181 (2)	
H5A	1.133568	0.761378	0.228996	0.022*	
H5B	0.976918	0.852922	0.162507	0.022*	
C6	0.96558 (12)	0.65077 (11)	0.27548 (9)	0.0157 (2)	
C7	0.80796 (12)	0.62521 (11)	0.42620 (9)	0.0158 (2)	
C8	0.66717 (12)	0.61609 (12)	0.57110 (9)	0.0192 (2)	
H8	0.615949	0.663132	0.615297	0.023*	
C9	0.72816 (11)	0.41325 (11)	0.53755 (9)	0.0156 (2)	
C10	0.80746 (11)	0.48500 (11)	0.44400 (9)	0.0149 (2)	
C11	0.88278 (11)	0.41606 (11)	0.36729 (9)	0.0145 (2)	
H11	0.949799	0.322367	0.414883	0.017*	
C12	0.76723 (11)	0.38847 (11)	0.29834 (9)	0.0150 (2)	
C13	0.74721 (12)	0.24974 (11)	0.31935 (9)	0.0167 (2)	
C14	0.63997 (13)	0.22961 (12)	0.25544 (10)	0.0203 (2)	
H14	0.626444	0.135374	0.270360	0.024*	
C15	0.55276 (13)	0.34813 (13)	0.16969 (10)	0.0219 (2)	
H15	0.479407	0.334475	0.126179	0.026*	
C16	0.57186 (12)	0.48584 (12)	0.14713 (9)	0.0202 (2)	
H16	0.512582	0.566631	0.088178	0.024*	
C17	0.67874 (12)	0.50423 (11)	0.21178 (9)	0.0166 (2)	
H17	0.691740	0.598756	0.196365	0.020*	
C18	0.95238 (14)	0.74386 (14)	0.01206 (10)	0.0250 (3)	
H18A	0.902460	0.845490	−0.015985	0.037*	
H18B	0.883323	0.683753	0.054365	0.037*	
H18C	0.984019	0.719560	−0.052170	0.037*	
C19	1.19764 (14)	0.80895 (13)	0.02337 (10)	0.0261 (3)	
H19A	1.229358	0.782730	−0.039966	0.039*	
H19B	1.284817	0.792140	0.073358	0.039*	
H19C	1.148514	0.910875	−0.006023	0.039*	
C20	0.66481 (12)	0.17663 (11)	0.65971 (9)	0.0161 (2)	
C21	0.54355 (12)	0.21974 (12)	0.71359 (10)	0.0191 (2)	
H21	0.495470	0.318317	0.686705	0.023*	
C22	0.49308 (13)	0.11803 (12)	0.80687 (10)	0.0213 (2)	
H22	0.411119	0.146464	0.844662	0.026*	
C23	0.56341 (13)	−0.02413 (12)	0.84358 (9)	0.0195 (2)	
C24	0.68061 (12)	−0.07101 (12)	0.79014 (9)	0.0192 (2)	
H24	0.725319	−0.170324	0.815788	0.023*	
C25	0.73138 (12)	0.03079 (12)	0.69795 (9)	0.0185 (2)	
H25	0.812624	0.000981	0.660219	0.022*	
C26	0.81945 (15)	−0.00492 (12)	0.42909 (11)	0.0276 (3)	
H26A	0.716838	−0.013407	0.447690	0.041*	
H26B	0.888194	−0.073905	0.493070	0.041*	
H26C	0.841765	−0.025339	0.361801	0.041*	

### 4-[(4-Fluorophenyl)amino]-5-(2-methoxyphenyl)-8,8-dimethyl-6,7,8,9-tetrahydro-5H-chromeno[2,3-d]pyrimidin-6-one Atomic displacement parameters (Å^2^)

**Table:**

	*U*^11^	*U*^22^	*U*^33^	*U*^12^	*U*^13^	*U*^23^
F1	0.0375 (4)	0.0219 (4)	0.0245 (4)	−0.0138 (3)	0.0131 (3)	−0.0072 (3)
O1	0.0237 (4)	0.0137 (4)	0.0192 (4)	−0.0056 (3)	0.0061 (3)	−0.0086 (3)
O2	0.0243 (4)	0.0199 (4)	0.0294 (4)	−0.0038 (3)	0.0068 (3)	−0.0145 (3)
O3	0.0245 (4)	0.0120 (4)	0.0229 (4)	−0.0032 (3)	−0.0011 (3)	−0.0078 (3)
N1	0.0222 (5)	0.0159 (4)	0.0191 (5)	−0.0034 (4)	0.0021 (4)	−0.0094 (4)
N2	0.0212 (5)	0.0176 (5)	0.0182 (4)	−0.0042 (4)	0.0031 (4)	−0.0090 (4)
N3	0.0219 (5)	0.0156 (4)	0.0172 (4)	−0.0051 (4)	0.0057 (4)	−0.0087 (4)
C1	0.0135 (5)	0.0170 (5)	0.0157 (5)	−0.0031 (4)	−0.0002 (4)	−0.0074 (4)
C2	0.0151 (5)	0.0196 (5)	0.0188 (5)	−0.0019 (4)	−0.0005 (4)	−0.0096 (4)
C3	0.0181 (5)	0.0230 (6)	0.0212 (5)	−0.0041 (4)	0.0052 (4)	−0.0107 (5)
C4	0.0203 (5)	0.0202 (5)	0.0168 (5)	−0.0054 (4)	0.0027 (4)	−0.0073 (4)
C5	0.0209 (5)	0.0170 (5)	0.0182 (5)	−0.0068 (4)	0.0018 (4)	−0.0075 (4)
C6	0.0148 (5)	0.0183 (5)	0.0158 (5)	−0.0034 (4)	0.0000 (4)	−0.0087 (4)
C7	0.0163 (5)	0.0157 (5)	0.0155 (5)	−0.0030 (4)	0.0002 (4)	−0.0065 (4)
C8	0.0218 (5)	0.0190 (5)	0.0195 (5)	−0.0027 (4)	0.0029 (4)	−0.0113 (4)
C9	0.0152 (5)	0.0159 (5)	0.0159 (5)	−0.0023 (4)	−0.0018 (4)	−0.0069 (4)
C10	0.0146 (5)	0.0151 (5)	0.0157 (5)	−0.0019 (4)	−0.0007 (4)	−0.0074 (4)
C11	0.0153 (5)	0.0126 (5)	0.0164 (5)	−0.0026 (4)	0.0012 (4)	−0.0069 (4)
C12	0.0143 (5)	0.0176 (5)	0.0163 (5)	−0.0037 (4)	0.0037 (4)	−0.0099 (4)
C13	0.0177 (5)	0.0166 (5)	0.0174 (5)	−0.0030 (4)	0.0037 (4)	−0.0088 (4)
C14	0.0227 (6)	0.0204 (5)	0.0237 (6)	−0.0084 (4)	0.0045 (4)	−0.0132 (5)
C15	0.0191 (5)	0.0297 (6)	0.0224 (6)	−0.0084 (5)	0.0015 (4)	−0.0146 (5)
C16	0.0167 (5)	0.0236 (6)	0.0190 (5)	−0.0018 (4)	0.0008 (4)	−0.0083 (5)
C17	0.0162 (5)	0.0163 (5)	0.0193 (5)	−0.0032 (4)	0.0043 (4)	−0.0092 (4)
C18	0.0274 (6)	0.0284 (6)	0.0190 (5)	−0.0050 (5)	−0.0015 (5)	−0.0097 (5)
C19	0.0286 (6)	0.0268 (6)	0.0232 (6)	−0.0099 (5)	0.0083 (5)	−0.0085 (5)
C20	0.0179 (5)	0.0165 (5)	0.0155 (5)	−0.0059 (4)	0.0001 (4)	−0.0070 (4)
C21	0.0183 (5)	0.0167 (5)	0.0225 (5)	−0.0031 (4)	0.0015 (4)	−0.0082 (4)
C22	0.0199 (5)	0.0229 (6)	0.0246 (6)	−0.0065 (4)	0.0069 (4)	−0.0125 (5)
C23	0.0236 (6)	0.0196 (5)	0.0178 (5)	−0.0111 (4)	0.0036 (4)	−0.0069 (4)
C24	0.0213 (5)	0.0154 (5)	0.0215 (5)	−0.0049 (4)	0.0000 (4)	−0.0073 (4)
C25	0.0177 (5)	0.0188 (5)	0.0213 (5)	−0.0042 (4)	0.0030 (4)	−0.0102 (4)
C26	0.0372 (7)	0.0137 (5)	0.0328 (7)	−0.0052 (5)	−0.0026 (5)	−0.0103 (5)

### 4-[(4-Fluorophenyl)amino]-5-(2-methoxyphenyl)-8,8-dimethyl-6,7,8,9-tetrahydro-5H-chromeno[2,3-d]pyrimidin-6-one Geometric parameters (Å, º)

**Table:**

F1—C23	1.3639 (13)	C11—C12	1.5339 (14)
O1—C6	1.3752 (13)	C12—C13	1.4015 (15)
O1—C7	1.3773 (13)	C12—C17	1.3875 (15)
O2—C2	1.2212 (14)	C13—C14	1.3923 (15)
O3—C13	1.3750 (13)	C14—H14	0.9500
O3—C26	1.4383 (13)	C14—C15	1.3903 (17)
N1—C7	1.3352 (14)	C15—H15	0.9500
N1—C8	1.3281 (14)	C15—C16	1.3842 (16)
N2—C8	1.3313 (14)	C16—H16	0.9500
N2—C9	1.3458 (14)	C16—C17	1.3869 (15)
N3—H3	0.8800	C17—H17	0.9500
N3—C9	1.3617 (14)	C18—H18A	0.9800
N3—C20	1.4163 (14)	C18—H18B	0.9800
C1—C2	1.4739 (14)	C18—H18C	0.9800
C1—C6	1.3396 (15)	C19—H19A	0.9800
C1—C11	1.5084 (14)	C19—H19B	0.9800
C2—C3	1.5107 (15)	C19—H19C	0.9800
C3—H3A	0.9900	C20—C21	1.3933 (15)
C3—H3B	0.9900	C20—C25	1.3964 (15)
C3—C4	1.5346 (16)	C21—H21	0.9500
C4—C5	1.5381 (15)	C21—C22	1.3911 (16)
C4—C18	1.5300 (16)	C22—H22	0.9500
C4—C19	1.5302 (15)	C22—C23	1.3741 (16)
C5—H5A	0.9900	C23—C24	1.3779 (16)
C5—H5B	0.9900	C24—H24	0.9500
C5—C6	1.4917 (14)	C24—C25	1.3851 (15)
C7—C10	1.3776 (15)	C25—H25	0.9500
C8—H8	0.9500	C26—H26A	0.9800
C9—C10	1.4161 (15)	C26—H26B	0.9800
C10—C11	1.5181 (14)	C26—H26C	0.9800
C11—H11	1.0000		
			
C6—O1—C7	118.15 (8)	C17—C12—C11	119.34 (9)
C13—O3—C26	117.23 (9)	C17—C12—C13	118.27 (10)
C8—N1—C7	113.92 (9)	O3—C13—C12	116.10 (9)
C8—N2—C9	116.04 (9)	O3—C13—C14	123.37 (10)
C9—N3—H3	115.5	C14—C13—C12	120.52 (10)
C9—N3—C20	128.94 (9)	C13—C14—H14	120.2
C20—N3—H3	115.5	C15—C14—C13	119.67 (10)
C2—C1—C11	117.68 (9)	C15—C14—H14	120.2
C6—C1—C2	118.84 (10)	C14—C15—H15	119.7
C6—C1—C11	123.44 (9)	C16—C15—C14	120.60 (10)
O2—C2—C1	120.09 (10)	C16—C15—H15	119.7
O2—C2—C3	122.08 (10)	C15—C16—H16	120.5
C1—C2—C3	117.79 (9)	C15—C16—C17	119.07 (10)
C2—C3—H3A	108.7	C17—C16—H16	120.5
C2—C3—H3B	108.7	C12—C17—H17	119.1
C2—C3—C4	114.23 (9)	C16—C17—C12	121.87 (10)
H3A—C3—H3B	107.6	C16—C17—H17	119.1
C4—C3—H3A	108.7	C4—C18—H18A	109.5
C4—C3—H3B	108.7	C4—C18—H18B	109.5
C3—C4—C5	107.76 (9)	C4—C18—H18C	109.5
C18—C4—C3	110.26 (9)	H18A—C18—H18B	109.5
C18—C4—C5	110.88 (9)	H18A—C18—H18C	109.5
C18—C4—C19	109.40 (9)	H18B—C18—H18C	109.5
C19—C4—C3	109.66 (9)	C4—C19—H19A	109.5
C19—C4—C5	108.85 (9)	C4—C19—H19B	109.5
C4—C5—H5A	109.1	C4—C19—H19C	109.5
C4—C5—H5B	109.1	H19A—C19—H19B	109.5
H5A—C5—H5B	107.8	H19A—C19—H19C	109.5
C6—C5—C4	112.67 (9)	H19B—C19—H19C	109.5
C6—C5—H5A	109.1	C21—C20—N3	123.85 (10)
C6—C5—H5B	109.1	C21—C20—C25	119.44 (10)
O1—C6—C5	111.10 (9)	C25—C20—N3	116.70 (9)
C1—C6—O1	123.39 (10)	C20—C21—H21	120.1
C1—C6—C5	125.51 (10)	C22—C21—C20	119.82 (10)
O1—C7—C10	122.57 (9)	C22—C21—H21	120.1
N1—C7—O1	111.73 (9)	C21—C22—H22	120.5
N1—C7—C10	125.69 (10)	C23—C22—C21	119.08 (10)
N1—C8—N2	128.12 (10)	C23—C22—H22	120.5
N1—C8—H8	115.9	F1—C23—C22	118.84 (10)
N2—C8—H8	115.9	F1—C23—C24	118.56 (10)
N2—C9—N3	118.94 (10)	C22—C23—C24	122.60 (10)
N2—C9—C10	121.92 (10)	C23—C24—H24	120.9
N3—C9—C10	119.13 (9)	C23—C24—C25	118.11 (10)
C7—C10—C9	114.28 (9)	C25—C24—H24	120.9
C7—C10—C11	122.25 (9)	C20—C25—H25	119.5
C9—C10—C11	123.45 (9)	C24—C25—C20	120.92 (10)
C1—C11—C10	108.98 (8)	C24—C25—H25	119.5
C1—C11—H11	109.0	O3—C26—H26A	109.5
C1—C11—C12	109.78 (8)	O3—C26—H26B	109.5
C10—C11—H11	109.0	O3—C26—H26C	109.5
C10—C11—C12	110.92 (8)	H26A—C26—H26B	109.5
C12—C11—H11	109.0	H26A—C26—H26C	109.5
C13—C12—C11	122.39 (9)	H26B—C26—H26C	109.5
			
F1—C23—C24—C25	−177.65 (9)	C8—N1—C7—C10	−0.57 (16)
O1—C7—C10—C9	179.51 (9)	C8—N2—C9—N3	178.18 (10)
O1—C7—C10—C11	−1.94 (16)	C8—N2—C9—C10	−1.16 (15)
O2—C2—C3—C4	151.38 (11)	C9—N2—C8—N1	−0.51 (17)
O3—C13—C14—C15	178.99 (10)	C9—N3—C20—C21	29.79 (17)
N1—C7—C10—C9	−0.85 (16)	C9—N3—C20—C25	−151.30 (11)
N1—C7—C10—C11	177.70 (10)	C9—C10—C11—C1	−171.70 (9)
N2—C9—C10—C7	1.76 (15)	C9—C10—C11—C12	67.33 (13)
N2—C9—C10—C11	−176.77 (9)	C10—C11—C12—C13	−109.02 (11)
N3—C9—C10—C7	−177.58 (9)	C10—C11—C12—C17	71.02 (12)
N3—C9—C10—C11	3.89 (15)	C11—C1—C2—O2	−3.57 (15)
N3—C20—C21—C22	−179.10 (10)	C11—C1—C2—C3	178.62 (9)
N3—C20—C25—C24	179.61 (10)	C11—C1—C6—O1	5.52 (16)
C1—C2—C3—C4	−30.86 (14)	C11—C1—C6—C5	−173.98 (10)
C1—C11—C12—C13	130.47 (10)	C11—C12—C13—O3	0.67 (14)
C1—C11—C12—C17	−49.48 (12)	C11—C12—C13—C14	179.36 (10)
C2—C1—C6—O1	−176.70 (9)	C11—C12—C17—C16	−179.60 (9)
C2—C1—C6—C5	3.80 (16)	C12—C13—C14—C15	0.40 (16)
C2—C1—C11—C10	170.48 (9)	C13—C12—C17—C16	0.44 (15)
C2—C1—C11—C12	−67.85 (11)	C13—C14—C15—C16	0.15 (17)
C2—C3—C4—C5	53.40 (12)	C14—C15—C16—C17	−0.40 (16)
C2—C3—C4—C18	−67.74 (12)	C15—C16—C17—C12	0.09 (16)
C2—C3—C4—C19	171.74 (9)	C17—C12—C13—O3	−179.37 (9)
C3—C4—C5—C6	−48.08 (12)	C17—C12—C13—C14	−0.68 (15)
C4—C5—C6—O1	−157.83 (9)	C18—C4—C5—C6	72.68 (12)
C4—C5—C6—C1	21.72 (15)	C19—C4—C5—C6	−166.94 (9)
C6—O1—C7—N1	174.52 (9)	C20—N3—C9—N2	−5.25 (17)
C6—O1—C7—C10	−5.79 (15)	C20—N3—C9—C10	174.11 (10)
C6—C1—C2—O2	178.52 (10)	C20—C21—C22—C23	−0.64 (17)
C6—C1—C2—C3	0.71 (15)	C21—C20—C25—C24	−1.43 (16)
C6—C1—C11—C10	−11.72 (14)	C21—C22—C23—F1	178.23 (10)
C6—C1—C11—C12	109.95 (11)	C21—C22—C23—C24	−1.42 (18)
C7—O1—C6—C1	4.09 (15)	C22—C23—C24—C25	2.00 (17)
C7—O1—C6—C5	−176.35 (9)	C23—C24—C25—C20	−0.54 (16)
C7—N1—C8—N2	1.36 (17)	C25—C20—C21—C22	2.02 (16)
C7—C10—C11—C1	9.88 (14)	C26—O3—C13—C12	−179.23 (10)
C7—C10—C11—C12	−111.09 (11)	C26—O3—C13—C14	2.12 (15)
C8—N1—C7—O1	179.10 (9)		

### 4-[(4-Fluorophenyl)amino]-5-(2-methoxyphenyl)-8,8-dimethyl-6,7,8,9-tetrahydro-5H-chromeno[2,3-d]pyrimidin-6-one Hydrogen-bond geometry (Å, º)

**Table:**

*D*—H···*A*	*D*—H	H···*A*	*D*···*A*	*D*—H···*A*
N3—H3···O3	0.88	2.08	2.9579 (13)	172
C15—H15···F1^i^	0.95	2.62	3.2998 (16)	129
C24—H24···O2^ii^	0.95	2.39	3.1930 (16)	141

Symmetry codes: (i) −*x*+1, −*y*, −*z*+1; (ii) −*x*+2, −*y*, −*z*+1.

## References

[bb1] Bruker (2009). *APEX2*, *COSMO* and *SAINT*. Bruker AXS Inc., Madison, Wisconsin, USA.

[bb2] Dolomanov, O. V., Bourhis, L. J., Gildea, R. J., Howard, J. A. K. & Puschmann, H. (2009). *J. Appl. Cryst.* **42**, 339–341.

[bb3] Krause, L., Herbst-Irmer, R., Sheldrick, G. M. & Stalke, D. (2015). *J. Appl. Cryst.* **48**, 3–10. 10.1107/S1600576714022985 PMC4453166 26089746

[bb4] Noser, A. A., Khalifa, M. M., Salman, I. N. & Salem, M. M. (2026). *Chem. Pap* **11**, 1–23.

[bb5] Patil, S. B. (2023). *Heliyon* **9**, e16773. 10.1016/j.heliyon.2023.e16773 PMC10279829 37346348

[bb6] Sheldrick, G. M. (2015*a*). *Acta Cryst.* A**71**, 3–8.

[bb7] Sheldrick, G. M. (2015*b*). *Acta Cryst.* C**71**, 3–8.

[bb8] Zamisa, S. J., Adeleke, A. A., Devnarain, N., Rhman, M. A., Owira, P. M. & Omondi, B. (2023). *RSC Adv.* **13**, 21820–21837. 10.1039/d3ra01741a PMC10354499 37475760

